# Identification of Two New Isolates of *Chilli veinal mottle virus* From Different Regions in China: Molecular Diversity, Phylogenetic and Recombination Analysis

**DOI:** 10.3389/fmicb.2020.616171

**Published:** 2020-12-23

**Authors:** Shaofei Rao, Xuwei Chen, Shiyou Qiu, Jiejun Peng, Hongying Zheng, Yuwen Lu, Guanwei Wu, Jianping Chen, Wen Jiang, Yachun Zhang, Fei Yan

**Affiliations:** ^1^State Key Laboratory for Managing Biotic and Chemical Threats to the Quality and Safety of Agro-products, Key Laboratory of Biotechnology in Plant Protection of Ministry of Agriculture and Zhejiang Province, Institute of Plant Virology, Ningbo University, Ningbo, China; ^2^Biotechnology Research Institute, Guangxi Academy of Agricultural Sciences, Nanning, China; ^3^Dali Bai Autonomous Prefecture Academy of Agricultural Science and Extension, Dali, China

**Keywords:** *Chilli veinal mottle virus*, phylogenetic analysis, recombination, genetic diversity, evolution

## Abstract

*Chilli veinal mottle virus* (ChiVMV) is an important plant pathogen with a wide host range, causing serious yield losses in pepper production all over the world. Recombination is a major evolutionary event for single-stranded RNA viruses, which helps isolates adapt to new environmental conditions and hosts. Recombination events have been identified in multiple potyviruses, but so far, there have been no reports of recombination events among the ChiVMV population. We here detected ChiVMV in pepper samples collected from Guangxi and Yunnan provinces for the first time and amplified the nearly full-length sequences. Phylogenetic and recombination analysis were performed using the new sequences and the 14 full-length and 23 capsid protein (CP) sequences available in GenBank. Isolates tend to cluster on a geographical basis, indicating that geographic-driven evolution may be an important determinant of ChiVMV genetic differences. A total of 10 recombination events were detected among the ChiVMV sequences using RDP4 with a strict algorithm, and both the Guangxi and Yunnan isolates were identified as recombinants. Recombination appears to be a significant factor affecting the diversity of ChiVMV isolates.

## Introduction

*Chilli veinal mottle virus* (ChiVMV) is a member of the genus *Potyvirus* in the family *Potyviridae*. It is a very common virus in chilli pepper (*Capsicum annum* L.) in east Asian countries, causing serious losses in pepper production (Ong et al., 1980; Moury et al., 2005; Wang et al., 2006; Tsai et al., 2008; Shah et al., 2009). In addition to *Capsicum annuum*, ChiVMV can also infect many other plants in the family *Solanaceae*, including *Nicotiana tabacum*, *Solanum lycopersicum*, *Solanum melongena*, and *Datura stramonium* (Tsai et al., 2008; Ding et al., 2011; Yang et al., 2013; Zhao et al., 2014; Kaur et al., 2015). Symptoms of ChiVMV infection include mosaic mottling, twisted or fallen leaves, vein banding, and reduced fruit size (Tsai et al., 2008; Gao et al., 2016). The genome of ChiVMV is a single-stranded sense RNA, about 9.7 kb excluding the poly (A) tail. It encodes a polyprotein, which is then cleaved by virus-encoded proteases into 10 mature functional proteins (Yang et al., 2013; Gao et al., 2016). *Aphis gossypii* has been reported to transmit the virus in a non-persistent manner in solanaceous crops (Shah et al., 2008).

The public sequence databases currently contain the complete genome sequences of 14 ChiVMV isolates, all of which are from Asia. Several studies have reported genetic differences within the species based on analysis of CP sequences (Moury et al., 2005; Yang et al., 2013; Gao et al., 2016; Ahmad and Ashfaq, 2018) and there are currently 98 such sequences available. Discovering and determining the sequences of more isolates worldwide is important for our understanding of the molecular diversity and evolution of the virus. For single-stranded RNA viruses, recombination is a major evolutionary event that helps isolates adapt to new environmental conditions and hosts (Simon-Loriere and Holmes, 2011). Recombination events have been identified in many potyviruses (Revers et al., 1996; Gagarinova et al., 2008; Seo et al., 2009) but, so far, there have been no reports of recombination events among the ChiVMV population. In this study, we determined the nearly full-length sequences of ChiVMV in pepper from Guangxi and Yunnan provinces, China and used the data to analyze molecular diversity and recombination events among ChiVMV isolates.

## Materials and Methods

### Whole-Genome Sequencing of Two New ChiVMV Isolates

From May to July 2020, we collected pepper samples with suspected viral disease symptoms (including dead tops, mosaic, mottling, wrinkled leaves, and chlorosis) from pepper fields in Guangxi and Yunnan provinces in China. Total RNA was extracted from infected pepper fruits using the Trizol method, and first strand cDNA was synthesized using a reverse transcription kit (Toyobo) according to the manufacturer’s instructions. The complete genome sequences of the two new isolates were amplified from five over-lapping fragments using specific primers (Supplementary Table 1). KOD neo enzyme (Toyobo) was used for PCR amplification, and the amplified fragments were purified with an Omega gel extraction kit and cloned into the pEASY-Blunt Zero vector (TransGen). At least two clones for each fragment were picked and sent for sequencing. Sequences were assembled using Vector NTI version 10. The complete genome sequences of the two isolates have been deposited in GenBank under accession numbers MT782116 (Guangxi) and MT974520 (Yunnan). Sequence analysis and comparison of the two new isolates to the other reported sequences were done using MEGA X (Kumar et al., 2018). The complete genome sequences and CP sequences of other ChiVMV isolates were downloaded from the National Center for Biotechnology Information (NCBI) (Supplementary Tables 2, 3).

### Construction of Phylogenetic Trees

The whole genome sequences of 16 ChiVMV isolates and 25 CP-coding region sequences were used for phylogenetic analysis in the MEGA X software package (Kumar et al., 2018). The best-fit nucleotide substitution models for the full-length sequences of 16 isolates and the 25 CP sequences were determined using the function in MEGA X to be, respectively, GTR + G + I (General Time Reversible + Gama Distributed With Invariant Sites) and T92 + G (Tamura 3-parameter + Gama Distributed). The trees were then constructed by the maximum-likelihood (ML) method according to the corresponding model with 1,000 bootstrap replicates. The sequence of the OKP41 isolate of pepper vein mottle virus (PVMV), a closely-related member of the genus *Potyvirus*, was used as an outgroup.

### Recombination and Selection Pressure Analysis

The whole genome sequences of 16 ChiVMV isolates and 25 CP-coding region sequences were used for recombination analysis. The six methods in the RDP4 software, namely RDP, GENECONV, BOOTSCAN, MaxChi, Chimera, and SISCAN were used to find possible parental isolates and recombination breakpoints with the default parameters (Martin et al., 2015). Site specific selection pressure in CP coding sequences, was determined by single likelihood ancestor counting (SLAC), fixed effects likelihood (FEL), mixed effects model of evolution (MEME) with *p-*value threshold of 0.1, and fast unconstrained Bayesian approximation (FUBAR) with posterior probability of 0.9 implemented in the Hyphy package^1^ (Pond and Frost, 2005).

## Results

### Sequencing and Molecular Diversity Analysis of ChiVMV Isolates From Guangxi and Yunnan

By using specific primers, we amplified overlapping fragments by RT-PCR, and assembled the nearly complete genome sequences of the ChiVMV Guangxi and Yunnan isolates. The sequences were 9,722 (Guangxi) and 9,724 (Yunnan) nucleotides long excluding the 3′-end poly A tail, and both contained a predicted open reading frame of 9,270 nt, encoding a poly-protein of 3,089 amino acids. In comparisons of their full-length genome sequences, the new isolates had 79–92.6% (Guangxi) and 80.6–89.8% (Yunnan) nt identity to the previously reported isolates while the corresponding values for comparison with the outgroup PVMV control sequence were, respectively, 67.4 and 68% (Table 1 and Supplementary Table 4). The divergence of the Guangxi isolate is about 0.08–0.25 and that of the Yunnan isolate is about 0.11–0.23 (Table 1 and Supplementary Table 4).

**TABLE 1:** Nucleotide sequence similarity and divergence among the 16 ChiVMV isolates worldwide based on their full genome sequences.

	MT782116 GX		MT974520 YN	
	Percent similarity	Percent divergence	Percent similarity	Percent divergence
KU987835.1 GD	92.5	8	81.9	21.1
KR296797.1 HN	92	8.5	81.5	21.5
JX088636.1 YN tobacco	79	25.1	82.6	20.3
GQ981316.1 Wenchang	92.6	7.9	81.9	21.2
AJ972878.1 Ca	92.1	8.2	81.3	21.8
KC711055.1 Yp8	81.7	21.4	89.8	11.1
KC711056.1 Pp4	81.8	21.1	89.6	11.3
GU170808.1 Ch-War	85.2	16.8	82.2	20.5
GU170807.1 Ch-Jal	85.4	16.5	82.2	20.6
AM909717.1 Korea	92.2	8.2	81.4	21.8
MN207122.1 PK	88.1	13	83.4	18.8
MK405594.1 SichuanLuzhou	81.7	21.6	89.7	11.2
NC_005778.1	85.7	16	80.6	22.8
LN832362.1 hn	92.1	8.3	81.5	21.5
MT782116 GX	–	–	81.8	21.3
MT974520 YN	81.8	21.3	–	–
LC438545.1 OKP41	67.4	42.9	68	42.2

*The values were calculated by DNAStar. The four columns of values represent the percent of nucleotide sequence similarity and divergence between the Guangxi and Yunnan isolates and each of the 14 isolates previously reported. See Supplementary Table 4 for other pairwise comparison values. The geographical origin of the isolates listed are: KU987835.1 GD, Guangdong, China; KR296797.1 HN, Hunan, China; JX088636.1 YN tobacco, Yunnan, China; GQ981316.1 Wenchang, Hainan, China; AJ972878.1 Ca, Korea; KC711055.1 Yp8, Sichuan, China; KC711056.1 Pp4, Sichuan, China; GU170808.1 Ch-War, India; GU170807.1 Ch-Jal, India; AM909717.1 Korea, Korea; MN207122.1 PK, Pakistan; MK405594.1 SichuanLuzhou, Sichuan, China; NC_005778.1, India; LN832362.1 hn, Hunan, China; MT782116 GX, Guangxi, China; MT974520 YN, Yunnan, China. LC438545.1 is an isolate of pepper vein mottle virus (PVMV).*

### Phylogenetic Relationships of ChiVMV Isolates Worldwide

**FIGURE 1:**
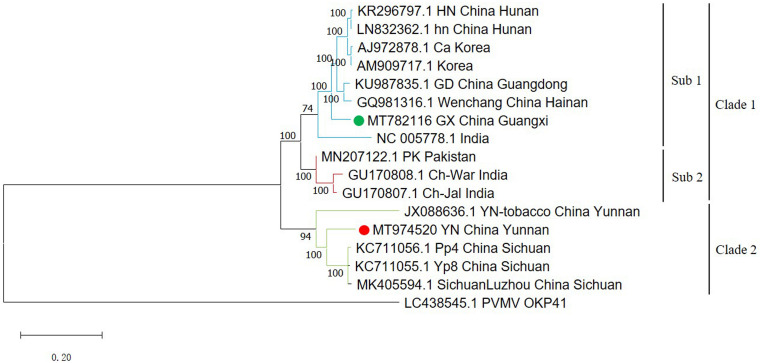
Phylogenetic tree of 16 full-length ChiVMV isolates. The phylogenetic tree of 16 full-length ChiVMV isolates was produced using MEGA X by the maximum-likelihood (ML) method using the General Time Reversible algorithm and 1,000 bootstrap replications. The ChiVMV isolates are divided into three sub-clades which are shown in different colors. The new Guangxi and Yunnan isolates are marked by green and red circles, respectively. The number at each branch of the phylogenetic tree is the bootstrap percentage. A pepper veinal mottle virus isolate sequence (LC438545) was used as outgroup.

A phylogenetic tree using all available full-length ChiVMV sequences with PVMV as an outgroup divides the isolates into two major clades (Figure 1). The first major clade has a sub-clade containing two isolates each from Hunan and Korea, and one isolate each from Guangdong, Wenchang, Guangxi (our isolate), and India. The second sub-clade is formed by two Indian and one Pakistani isolates. The second major clade contains three Sichuan isolates and one Yunnan tobacco isolate (Figure 1). The newly identified Yunnan pepper isolate was also included in the second clade. Thus our Guangxi and Yunnan isolates were more similar to the isolates from adjacent regions of China (such as Wenchang, Guangdong for Guangxi isolate, and Sichuan for Yunnan isolate), than to those from the more distant provinces.

**FIGURE 2:**
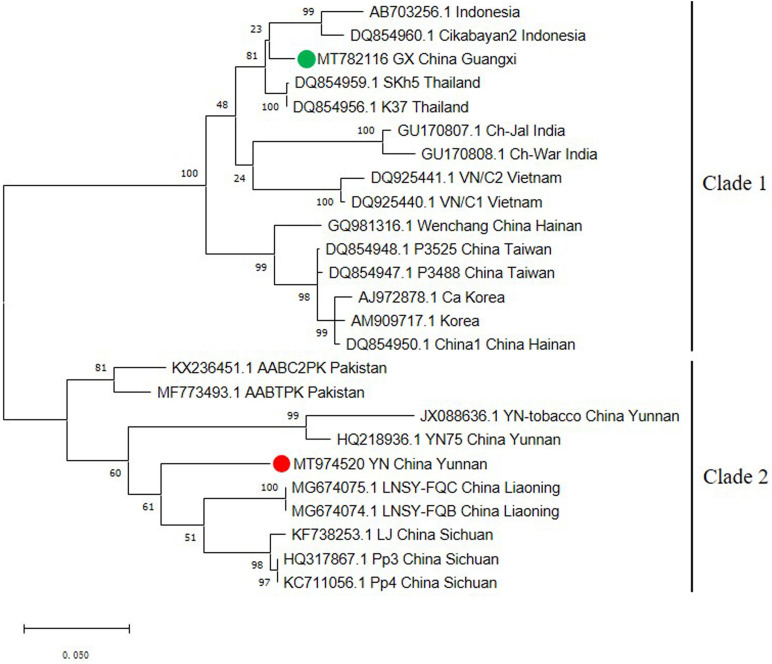
Phylogenetic tree based on the nucleotide sequences of the CP gene of 25 ChiVMV isolates. The phylogenetic tree was produced using MEGA X by the maximum-likelihood (ML) method using the Tamura 3-parameter algorithm and 1,000 bootstrap replications. The number at each branch of the phylogenetic tree is the bootstrap percentage. The tree was divided into two clades and the new Guangxi and Yunnan isolates are marked by green and red circles, respectively.

The capsid protein (CP) gene is very important for the infection cycle of potyviruses. Its primary function is to encode the virus coat protein (Revers and García, 2015) and this region has often been used as the basis for comparisons to establish the taxonomy of potyviruses (Ball, 2005; Tsai et al., 2008). To better understand the genetic variability of the ChiVMV population, we selected 25 CP coding region sequences from different geographical locations to construct a phylogenetic tree. The tree had two well-defined major clades (Figure 2). The first clade contains isolates from Indonesia, Thailand, India, Vietnam, South Korea and China (Guangxi, Hainan and Taiwan). Isolates from Yunnan, Liaoning, Sichuan of China and Pakistan constitute the second clade (Figure 2).

### Recombination and Selection Pressure Analysis

To analyze possible recombination signals in the ChiVMV population, we used RDP4 software to predict recombination events among the full-length sequences. Recombination events identified by at least three methods and *P* value less than 1 × 10^–6^ were considered credible and 10 recombination events were detected in total (Table 2). The Guangxi and Yunnan isolates were predicted to be recombinants. The recombination event of the Guangxi isolate occurred between nts 2,756 and 5,284. The Hunan isolate and the Wenchang (Hainan Province) isolate were predicted to be possible parents. Interestingly, Hunan and Hainan are both neighboring provinces to Guangxi. The recombination of Yunnan isolate occurred between nts 31 and 1,400, and Pakistan isolate and Yunnan tobacco isolate were predicted as possible parents (Table 2). The analysis indicated that geography-related recombination was likely a key factor in the evolution of ChiVMV.

**TABLE 2:** Summary of possible recombination events among 16 full-length ChiVMV isolates identified by RDP4.

Event number	Begin	End	Recombinant sequence(s)	Minor parental sequence(s)	Major parental sequence(s)	*P*-value for the six detection methods in RDP4					
						RDP	GENECONV	Bootscan	Maxchi	Chimaera	SiSscan
1	2756	5284	MT782116 GX	GQ981316.1 Wenchang	Unknown (LN832362.1_hn)	3.55E-07	NS	4.92E-06	5.46E-08	1.34E-08	6.53E-07
2	31	1400	MT974520 YN	JX088636.1 YN tobacco	MN207122.1 PK	2.20E-44	4.85E-21	1.32E-38	3.83E-05	5.10E-17	1.53E-34
3	9316	9739	JX088636.1 YN tobacco	KC711055.1_Yp8	Unknown (MN207122.1 PK)	8.99E-06	NS	1.95E-06	NS	NS	2.23E-08
4	27	1696	JX088636.1 YN tobacco	MK405594.1 SichuanLuzhou	Unknown (GQ981316.1 Wenchang)	1.01E-25	4.01E-18	8.95E-22	8.12E-13	1.07E-09	8.80E-34
5	5604	5854	GU170808.1 Ch-War	Unknown (MN207122.1 PK)	GU170807.1 Ch-Jal	1.29E-19	7.58E-17	9.33E-20	2.27E-10	2.56E-09	1.56E-13
6	3035	4198	GU170807.1 Ch-Jal	KC711055.1_Yp8	AM909717.1 Korea	2.15E-10	1.72E-03	1.02E-10	2.69E-06	1.91E-07	4.29E-15
7	5658	8580	NC-005778.1 India	Unknown (GU170807.1 Ch-Jal)	AM909717.1 Korea	1.77E-09	NS	3.09E-09	2.00E-13	7.24E-07	9.45E-27
8	2401	2822	MN207122.1 PK	KC711056.1_Pp4	LN832362.1_hn	9.66E-13	1.24E-09	1.38E-12	2.05E-08	2.23E-05	1.22E-11
9	1894	4480	MN207122.1 PK	KR296797.1_HN	GU170808.1 Ch-War	4.93E-06	2.59E-05	8.94E-06	2.40E-10	3.35E-10	NS
10	27	1389	MN207122.1 PK	Unknown (JX088636.1 YN tobacco)	MK405594.1 SichuanLuzhou	1.21E-34	1.80E-20	1.37E-33	1.74E-14	9.05E-17	1.28E-34

*The whole genome sequences of the two newly identified isolates and 14 ChiVMV full-length sequences extracted from public nucleic acid databases were used for recombination analysis. The six methods in the RDP4 software, namely RDP, GENECONV, BOOTSCAN, MaxChi, Chimaera, and SISCAN were used to find possible parental isolates and recombination breakpoints with the default parameters. Recombination events identified by at least three methods and *P* value less than 1 × 10^–6^ are listed. For the geographical origin of the isolates, see Table 1. NS, Not Significant.*

We also detected two recombination events in the Yunnan tobacco isolate, at nt positions 9,316–9,739 and 27–1,696, respectively. One recombination event was detected in each of the three Indian isolates, GU170808, GU170807, and NC_005778. Three recombination events were detected in the MN207122-PK isolate, at various sites in the region 27–4,480 (Table 2).

Whether there were recombination events in the 25 selected CP sequences from different geographical locations was further analyzed, and 3 recombination events were found (Supplementary Table 5). Two Chinese isolates and one Pakistan isolate were predicted to be recombinants, indicating that the recombination contributed to the variation of the CP sequences (Supplementary Table 5).

We also performed selection pressure analysis on the CP coding sequences (287 aa), and found that many of the codons are subject to negative selection. A total of 87, 136, and 160 negatively selected codons were detected in the CP region by SLAC, FEL, and FUBAR methods, respectively. The codons 83, 173, 176, 231, 239, 261, and 268 were found to be under positive selection by MEME.

## Discussion

In this study, we detected ChiVMV in pepper disease samples collected from Guangxi and Yunnan provinces for the first time, and amplified the nearly full-length sequences of these isolates by overlapping PCR. Among the other complete ChiVMV sequences that have been made available, Guangxi isolate was least similar (79% nt identity) to the Yunnan tobacco isolate suggesting that the host imposes a selective effect on the evolution of the virus (Table 1). However, since the Yunnan tobacco isolate was predicted to be a minor parent of the Yunnan pepper isolate identified in our work (Table 2), the similarity between these two was slightly higher. The Yunnan pepper isolate has the lowest nt similarity with an isolate from India, with a value of 80.6% (Table 1). Published studies of the evolution and variation of ChiVMV isolates have usually been based on CP sequences (Moury et al., 2005; Tsai et al., 2008; Yang et al., 2013; Gao et al., 2016; Ahmad and Ashfaq, 2018). By constructing a phylogenetic tree from the full-length sequences, we found that the viral isolates from the same or similar regions tend to group together (Figure 1). The analysis suggests that the evolutionary adaptation of the virus is driven by geographic location, which is consistent with the conclusion of Gao et al. (2016). However, in addition to Chinese isolates, we found that there were isolates from South Korea and India that were in the same clade as the Guangxi isolate (Figure 1). This may be due to the frequent trade of vegetables and ornamentals between China and these countries.

Recombination is considered to be a significant source of plant virus genetic diversity (Simon-Loriere and Holmes, 2011). Recombination events have been identified in several potyviruses, including soybean mosaic virus, potato virus Y etc., (Revers et al., 1996; Gagarinova et al., 2008; Seo et al., 2009). Our analysis detected a total of 10 recombination events within the full-length ChiVMV sequences, and the newly identified Guangxi and Yunnan isolates were both recognized as recombinants (Table 2), indicating that recombination plays an important role in shaping the adaptability of the ChiVMV population. We also detected three recombination events among 25 selected CP sequences from different geographical locations, and the Guangxi isolate was predicted to be a minor parent of the Sichuan isolate (KF738253.1) (Supplementary Table 5).

The site-specific selection pressure analysis of the CP coding region using a variety of methods shows that codons at many sites are subject to negative selection, which is consistent with previous findings (Ahmad and Ashfaq, 2018), suggesting a strong negative or purifying selection in the ChiVMV population.

In conclusion, our study has determined the nearly complete genome sequences of two ChiVMV isolates from Guangxi and Yunnan provinces, China. Comparisons with other sequences have shown that the genetic differences among ChiVMV isolates were likely correlated with geographical location. Recombination occurs actively in the ChiVMV population and may be a force driving the adaptive evolution of the virus. A comparative analysis of the genome sequences of additional ChiVMV isolates would be helpful to give a clearer picture of genetic variability and evolution in this important virus.

## Data Availability Statement

The datasets presented in this study can be found in online repositories. The names of the repository/repositories and accession number(s) can be found in the article/Supplementary Material.

## Author Contributions

SR and FY: conceptualization. JC and FY: funding acquisition. SR, XC, and SQ: investigation. SR, JP, HZ, YL, and GW: methodology. WJ and YZ: resources. FY: supervision. SR: writing – original draft. FY: writing – review and editing. All authors contributed to the article and approved the submitted version.

## Conflict of Interest

The authors declare that the research was conducted in the absence of any commercial or financial relationships that could be construed as a potential conflict of interest.

## Footnotes

1 http://www.Datamonkey.org
