# Corrigendum: Identification of Two New Isolates of *Chilli veinal mottle virus* From Different Regions in China: Molecular Diversity, Phylogenetic and Recombination Analysis

**DOI:** 10.3389/fmicb.2021.670393

**Published:** 2021-04-19

**Authors:** Shaofei Rao, Xuwei Chen, Shiyou Qiu, Jiejun Peng, Hongying Zheng, Yuwen Lu, Guanwei Wu, Jianping Chen, Wen Jiang, Yachun Zhang, Fei Yan

**Affiliations:** ^1^State Key Laboratory for Managing Biotic and Chemical Threats to the Quality and Safety of Agro-products, Key Laboratory of Biotechnology in Plant Protection of Ministry of Agriculture and Zhejiang Province, Institute of Plant Virology, Ningbo University, Ningbo, China; ^2^Biotechnology Research Institute, Guangxi Academy of Agricultural Sciences, Nanning, China; ^3^Dali Bai Autonomous Prefecture Academy of Agricultural Science and Extension, Dali, China

**Keywords:** *Chilli veinal mottle virus*, phylogenetic analysis, recombination, genetic diversity, evolution

There is an error in the Funding statement. The correct Name for the Funder is ^**^Chinese Agriculture Research System (CARS-24-C-04) and K. C. Wong Magna Fund in Ningbo University^**^.

In the original article, there was an error. ^**^The recipient of the funding is incorrect^**^.

A correction has been made to ^**^Authors' contributions^**^:

^**^Funding acquisition, Jianping Chen and Fei Yan^**^

The authors apologize for this error and state that this does not change the scientific conclusions of the article in any way. The original article has been updated.

