# Supplementary material for: Identification of Two New Isolates of Chilli veinal mottle virus From Different Regions in China: Molecular Diversity, Phylogenetic and Recombination Analysis
Source: Front Microbiol. 2020 Dec 23;11:616171. doi: 10.3389/fmicb.2020.616171 (PMC7785935; doi:10.3389/fmicb.2020.616171)
Supplement: Supplementary file 1 [file Table_1.docx]

**Supplementary table 1 Primers used in this study.**

| **Primer name** | **Sequence** |
| --- | --- |
| ChiVMV F1 FOR | AATACAAACATACAGAAAACAAACGAAT |
| ChiVMV F1 REV | CCACCAACTCTRTACATYTTCATCTC |
| ChiVMV F2 FOR | TAGGRCAGTGGCCRACAATGACYG |
| ChiVMV F2 REV | ATTCCTTTATTYGTGTRMCCAATTCT |
| ChiVMV F3 FOR | ACAAAAGTTGATGGKAGGACRATGAA |
| ChiVMV F3 REV | CCTATTTCATTTGGRTTRTACACCC |
| ChiVMV F4 FOR | AACTCATCAACWATAGCTGGATTYCC |
| ChiVMV F4 REV | TCAGCTCTATCCCAYTCAAGRATYGA |
| ChiVMV F5 FOR | GCTGATGGRACAATAGTCAARAAGTT |
| ChiVMV F5 REV | AWCGCCAACTATTGAAYARTYYAMCG |
