# Supplementary material for: Identification of Two New Isolates of Chilli veinal mottle virus From Different Regions in China: Molecular Diversity, Phylogenetic and Recombination Analysis
Source: Front Microbiol. 2020 Dec 23;11:616171. doi: 10.3389/fmicb.2020.616171 (PMC7785935; doi:10.3389/fmicb.2020.616171)
Supplement: Supplementary file 2 [file Table_2.docx]

**Supplementary table 2** **Detailed information of the isolates with whole genome sequences used for analysis in this study.**

| **No.** | **Accession no.** | **isolate name** | **host** | **country** |
| --- | --- | --- | --- | --- |
| 1 | KU987835.1 | GD | pepper | China (Guangdong) |
| 2 | KR296797.1 | HN | *Capsicum annuum* | China (Hunan) |
| 3 | JX088636.1 | YN-tobacco | tobacco | China (Yunnan) |
| 4 | GQ981316.1 | Wenchang | *Capsicum annuum* | China (Hainan) |
| 5 | AJ972878.1 | Ca | Unknown | Korea |
| 6 | KC711055.1 | Yp8 | *Capsicum annuum* | China (Sichuan) |
| 7 | KC711056.1 | pp4 | *Capsicum annuum* | China (Sichuan) |
| 8 | GU170808.1 | ChiVMV-Ch-War | hot pepper | India |
| 9 | GU170807.1 | ChiVMV-Ch-Jal | hot pepper | India |
| 10 | AM909717.1 | Korea | pepper | Korea |
| 11 | MN207122.1 | ChiVMV-PK | *Capsicum annuum* | [Pakistan](javascript:;) |
| 12 | MK405594.1 | SichuanLuzhou | *Nicotiana tabacum* | China (Sichuan) |
| 13 | NC_005778.1 | None | *Capsicum annuum* | India |
| 14 | LN832362.1 | hn | Unknown | China (Hunan) |
| 15 | MT782116 | GX | *Capsicum annuum* | China (Guangxi) |
| 16 | MT974520 | YN | *Capsicum annuum* | China (Yunnan) |

All information were collected from the NCBI public database.
