# Supplementary material for: Identification of Two New Isolates of Chilli veinal mottle virus From Different Regions in China: Molecular Diversity, Phylogenetic and Recombination Analysis
Source: Front Microbiol. 2020 Dec 23;11:616171. doi: 10.3389/fmicb.2020.616171 (PMC7785935; doi:10.3389/fmicb.2020.616171)
Supplement: Supplementary file 3 [file Table_3.docx]

**Supplementary table 3 Detailed information of the isolates with CP sequences used for analysis in this study.**

| **No.** | **Accession no.** | **Isolate name** | **Host** | **Country** |
| --- | --- | --- | --- | --- |
| 1 | KF738253.1 | LJ | *Lycopersicon esculentum* | China (Sichuan) |
| 2 | AB703257.1 | None | *Capsicum annuum* | Indonesia |
| 3 | MG674075.1 | LNSY-FQC | *Solanum lycopersicum* | China (Liaoning) |
| 4 | MG674074.1 | LNSY-FQB | *Solanum lycopersicum* | China (Liaoning) |
| 5 | JX088636.1 | YN-tobacco | tobacco | China (Yunnan) |
| 6 | GU170807.1 | Ch-Jal | hot pepper | India |
| 7 | GQ981316.1 | Wenchang | *Capsicum chinense Jacp. cv. Yellow Lantern* | China (Hainan) |
| 8 | AM909717.1 | Korea | pepper | Korea |
| 9 | AJ972878.1 | Ca | Unknow | Korea |
| 10 | KX236451.1 | AABC2PK | *Capsicum sp. (hot chilli)* | Pakistan |
| 11 | HQ218936.1 | YN75 | *Nicotiana tabacum* | China (Yunnan) |
| 12 | DQ854960.1 | Cikabayan2 | *Capsicum sp.* | Indonesia |
| 13 | DQ854959.1 | SKh5 | *Capsicum sp.* | Thailand |
| 14 | DQ854956.1 | K37 | *Capsicum sp.* | Thailand |
| 15 | DQ854950.1 | China1 | *Capsicum sp.* | China (Hainan) |
| 16 | DQ854948.1 | P3525 | *Capsicum sp.* | China (Taiwan) |
| 17 | DQ854947.1 | P3488 | *Capsicum sp.* | China (Taiwan) |
| 18 | HQ317867.1 | Pp3 | chilli | China (Sichuan) |
| 19 | KC711056.1 | Pp4 | *Capsicum chinense Jacp.* | China (Sichuan) |
| 20 | GU170808.1 | Ch-War | hot pepper | India |
| 21 | MF773493.1 | AABTPK | tomato | Pakistan |
| 22 | DQ925446.1 | VN/C6 | *Capsicum annuum* | Vietnam |
| 23 | DQ925440.1 | VN/C1 | *Capsicum annuum* | Vietnam |
| 24 | MT782116 | GX | *Capsicum annuum* | China (Guangxi) |
| 25 | MT974520 | YN | *Capsicum annuum* | China (Yunnan) |

All information were collected from the NCBI public database.
