# Supplementary material for: Identification of Two New Isolates of Chilli veinal mottle virus From Different Regions in China: Molecular Diversity, Phylogenetic and Recombination Analysis
Source: Front Microbiol. 2020 Dec 23;11:616171. doi: 10.3389/fmicb.2020.616171 (PMC7785935; doi:10.3389/fmicb.2020.616171)
Supplement: Supplementary file 5 [file Table_5.docx]

**Supplementary Table 5 Summary of possible recombination events of 25 ChiVMV capsid protein (CP) coding region sequences identified by RDP4.**

| Event Number | Begin | End | Recombinant Sequence(s) | Minor Parental Sequence(s) | Major Parental Sequence(s) | P-Value for the six detection methods in RDP4 | | | | | |
| --- | --- | --- | --- | --- | --- | --- | --- | --- | --- | --- | --- |
|  |  |  |  |  |  | RDP | GENECONV | Bootscan | Maxchi | Chimaera | SiSscan |
| 1 | 410 | 856 | HQ218936.1 YN75 China Yunnan | GU170807.1 Ch-Jal India | Unknown (DQ854947.1 P3488 China Taiwan) | NS | NS | NS | NS | 3.92E-03 | 2.61E-08 |
| 2 | 420 | 706 | KX236451.1 AABC2PK Pakistan | GQ981316.1 Wenchang China Hainan | Unknown (GU170807.1 Ch-Jal India) | NS | NS | NS | 1.23E-02 | NS | 6.58E-08 |
| 3 | 486 | 861 | KF738253.1 LJ China Sichuan | MT782116 GX China Guangxi | MG674074.1 LNSY-FQB China Liaoning | 4.02E-02 | NS | NS | 6.35E-07 | 5.90E-05 | 1.75E-14 |

Note: 25 ChiVMV capsid protein (CP) coding region sequences extracted from public nucleic acid databases were used for recombination analysis. The six methods in the RDP4 software, namely RDP, GENECONV, BOOTSCAN, MaxChi, Chimaera and SISCAN were used to find possible parental isolates and recombination breakpoints with the default parameters. NS: Not Significant.
